# CD36 plays an important role in the clearance of oxLDL and associated age-dependent sub-retinal deposits

**DOI:** 10.18632/aging.100218

**Published:** 2010-11-09

**Authors:** Emilie Picard, Marianne Houssier, Kim Bujold, Przemyslaw Sapieha, William Lubell, Allison Dorfman, Julie Racine, Pierre Hardy, Maria Febbraio, Pierre Lachapelle, Huy Ong, Florian Sennlaub, Sylvain Chemtob

**Affiliations:** ^1^ Departments of Pediatrics, Ophthalmology, and Pharmacology, Research Center, Hospitals Ste. Justine and Maisonneuve-Rosemont, Université de Montréal, Montreal, Quebec, Canada; ^2^ Inserm, U872, Paris, F-75006 France; ^3^ Centre de Recherche des Cordeliers, Université Pierre et Marie Curie - Paris 6, UMR S 872, Paris, F-75006 France; ^4^ Université Paris Descartes, UMR S 872, Paris, F-75006 France; ^5^ Faculty of Pharmacy, University de Montreal, Montreal, Quebec, Canada; ^6^ Departments of Chemistry, University de Montreal, Montreal, Quebec, Canada; ^7^ Departments of Ophthalmology, McGill University, Montreal, Quebec, Canada; ^8^ Department of Cell Biology, Lerner Research Institute, Cleveland Clinic Foundation, Cleveland, OH 4412, USA; ^9^ APHP, Département d'Ophthalmologie Hôtel Dieu, Paris, France

**Keywords:** CD36, oxidized lipids, Bruch's membrane, age-related macular degeneration

## Abstract

Age-related macular degeneration (AMD) represents the major cause of vision loss in industrialized nations. Laminar deposits in Bruch's membrane (BM) are among the first prominent histopathologic features, along with drusen formation, and have been found to contain oxidized lipids. Increases in concentrations of oxidized LDL (oxLDL) in plasma are observed with age and high fat high (HFHC) cholesterol diet. CD36 is the principal receptor implicated in uptake of oxLDL, and is expressed in the retinal pigment epithelium (RPE). We determined if CD36 participates in oxLDL uptake in RPE and correspondingly in clearance of sub-retinal deposits. Uptake of oxLDL by RPE *in vitro* and *in vivo* was CD36-dependent. CD36 deficiency in mice resulted in age-associated accumulation of oxLDL and sub-retinal BM thickening, despite fed a regular diet. Conversely, treatment of HFHC-fed ApoE null mice with a CD36 agonist, EP80317 (300 μg/kg/day), markedly diminished thickening of BM, and partially preserved (in part) photoreceptor function. In conclusion, our data uncover a new role for CD36 in the clearance of oxidized lipids from BM and in the prevention of age-dependent sub-retinal laminar deposits.

## INTRODUCTION

Age-related macular degeneration (AMD) is the leading cause of vision loss among older adults in industrialized nations [1]. One of the first signs of AMD is the presence of drusen and extracellular basal deposits in Bruch's membrane (BM) [2], leading to BM thickening, and ultimately is associated with injury to the retinal pigmented epithelium (RPE), choroid and photoreceptors [3-5]. With age, esterified cholesterol associated in lipoprotein-like particles accumulate sub-retinally in BM deposits [6-10].

Lipids in the RPE mostly originate from spent photoreceptor outer segments, and are exocytosed through the base of the RPE to be eliminated via the choroid [11-13]. However, RPE cells also take-up oxidized lipids from their cell base [14, 15], suggesting they may be involved in clearing the sub-retinal space from such deposits [16]. Accumulation of lipoprotein- and lipid-containing sub-RPE and BM debris implies an imbalance in the accumulation/clearance of these products, leading to cytotoxicity [17,18]. Correspon-dingly, high fat cholesterol diet (HFHC)-fed mice deficient in low-density lipoprotein (LDL) receptor ligand ApoE (ApoE−/−) and rabbits on fat-enriched diet, feature high LDL and oxidized LDL (oxLDL) plasma levels [19-21] and exhibit ultrastructural retinal changes similar to those observed in human AMD [22], including BM thickening and BM lipid deposits [23-25]. These findings support the concept that improper clearance of dietary lipids may influence the development of AMD [26].

The RPE and microvascular endothelium contain a number of scavenger receptors involved in uptake and metabolism of oxidized lipids [27,28]. Of the various scavenger receptors CD36 is a principal receptor of oxidized phospholipid [15] expressed at the basolateral side of RPE as well as on microvascular endothelial cells and macrophages [29-33]. Interestingly, a polymorphism of CD36 gene (in the non-coding region [possibly associated with its increased expression]) is protective against AMD [34]. We previously demonstrated that mice with CD36 gene disruption revealed a progressive age-dependent photoreceptor death and choroidal involution [32]. We hereby hypothesized that CD36 participates in oxLDL uptake in RPE and correspondingly in (clearance of) sub-retinal deposits. Our findings reveal that uptake of oxLDL in RPE is CD36-dependent and that a deficiency in CD36 leads to accumulation of sub-retinal deposits and oxLDL, despite fed a regular diet; conversely, stimulation of CD36 reduces sub-retinal deposits and preserves photoreceptor function.

**Figure 1.**
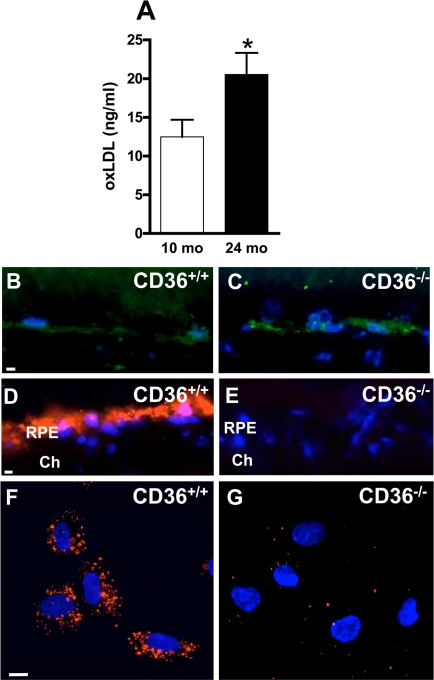
CD36 deficiency inhibits oxLDL uptake by RPE cells and leads to oxLDL accumulation (**A**) oxLDL concentrations in plasma increase with normal aging. Immuno-localization of oxLDL (green) on frozen sections of retina from 12 months (mo)-old CD36+/+ (**B**) and CD36−/−. (**C**) mice. *In vivo* incorporation of fluorescent DiI-tagged oxLDL (red) by retinal pigment epithelium (RPE) administered intravenously in 2-month-old CD36+/+ (**D**) and CD36−/− mice (**E**). *In vitro* uptake of DiI-oxLDL by RPE from CD36+/+ mice (F) or CD36−/− mice (**G**). Cells and sections were counterstained with Dapi (blue). Ch Choriocapillaris. Scale bar: 10μm. Values are mean ± SEM; n=4-6/group. * p<0.05 compared to values without asterisks.

## RESULTS

### oxLDL uptake by RPE cells is CD36-dependent

Blood levels of oxLDL increase with age [40] and upon ingestion of high cholesterol diet [20], as corroborated (Figure 1A). Since CD36 clears circulating oxLDL [29], we tested if CD36 deficiency would lead to accumulation of oxLDL in the sub-retinal region. CD36-deficient mice presented abundant sub-retinal oxLDL accumulation (Figure 1B,C), despite regular diet; oxLDL was minimally detected in comparably raised wild type (WT, CD36+/+) congeners. CD36-dependent cellular uptake of oxLDL was confirmed upon systemic administration of fluorescent tagged oxLDL; specific uptake of DiI-oxLDL was seen in the RPE of CD36+/+ mice (Figure 1D), whereas no fluorescence was detected in RPE of CD36−/− mice in vivo (Figure 1E). Likewise, exposure of RPE cells from CD36+/+ mice to DiI-oxLDL revealed internalization of the oxLDL, while no internalization was seen in RPE of CD36−/− mice (Figure 1F,G).

**Figure 2.**
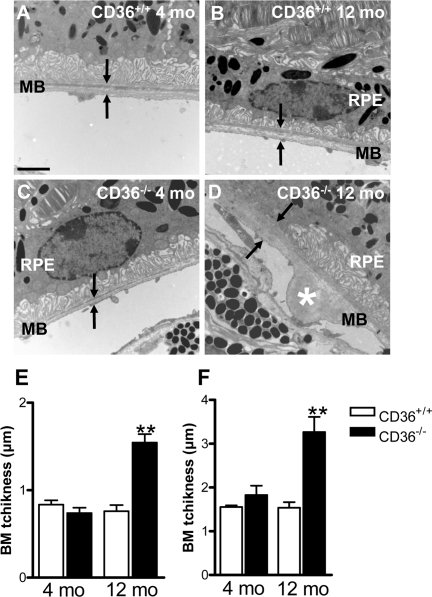
CD36 deficiency leads to BM thickening Transmission electron microscopy of RPE/sub-RPE region in 4 months (mo)-old CD36+/+ (**A**) and CD36−/− (**C**) showed similar BM thickness; arrowhead points to Bruch's membrane (BM). While, 12-month-old CD36+/+ (**B**) and CD36−/− mice (**D**) reveal increased BM thickness and nodular debris (asterix) in BM of CD36−/− subjects (**D**). BM average (**E**) and maximal (**F**) thickness in CD36+/+ and CD36−/− mice at 4 and 12 months of age. RPE refers to retinal pigment epithelium. Scale bar: 1μm. Values are mean ± SEM; n=5/group. * p<0.01 compared to values without asterisks.

### CD36 deficiency leads to BM thickening

Lipids are the main component in basal laminar deposits [8,13,41]. We evaluated if sub-RPE accumulation of oxLDL in CD36−/− mice (Figure 1C) is associated with BM thickening. Electron microscopy of the sub-retinal region revealed age-dependent debris detected in older (12 months-old) but not younger (4 months-old) CD36−/− mice (Figure 2A-F).

**Figure 3.**
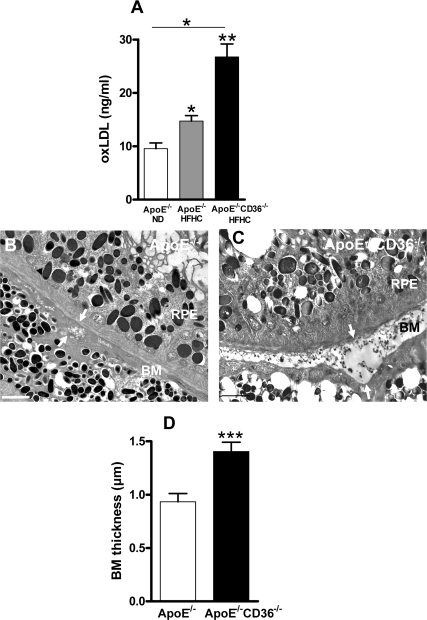
CD36 deficiency enhances BM thickness in high fat high cholesterol fed ApoE−/− mice (**A**) Plasma concentration of oxLDL in 4 months-old ApoE−/− mice under normal diet (ND) or high fat high cholesterol (HFHC) diet, and in double knockout ApoE−/−CD36−/− mice under HFHC diet was evaluated by ELISA. Transmission electron microscopy of Bruch's membrane (BM) thickening (arrows) in 4 months-old ApoE−/− (**B**) and ApoE−/−CD36−/− mice (**C**) both under HFHC diet. (**D**) Compiled BM thickness in 4-month-old ApoE−/− and ApoE−/−CD36−/− mice. Scale bars: 2μm. RPE refers to retinal pigmented epithelium. Values are mean ± SEM; n=4-6/group. *p<0.05, ** p<0.01, ***p<0.001, compared to values without asterisks.

Mice deficient in the LDL receptor ligand, ApoE (ApoE−/−) involved in uptake of LDL, fed a HFHC diet, exhibit an atherosclerosis phenotype with high blood oxLDL levels [21]; along the lines of the concept presented herein, these animals also exhibit laminar deposits in BM, as seen in AMD [24]. To further address the role of CD36 on oxLDL accumulation and BM thickness, oxLDL plasma levels and electron microscopy of the sub-retinal region were performed in ApoE−/− mice and in ApoE/CD36 double knockout mice (ApoE−/−/CD36−/−). oxLDL levels were highest in the 4 months-old HFHC-fed ApoE/CD36 double knockout mice (Figure 3A). In addition, BM thickening was further augmented in these relatively young ApoE−/−/CD36−/− mice (Figure 3B-D). Collectively, data indicate that CD36 deficiency is associated with augmented sub-retinal deposits.

**Figure 4.**
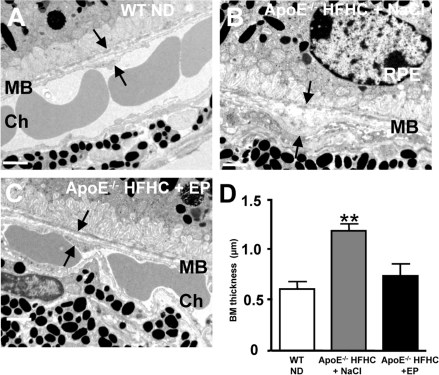
CD36 stimulation reduces BM thickness in high cholesterol fed ApoE −/− mice Transmission electron microscopy of Bruch's membrane (BM) thickening (arrows) in WT mice under ND (**A**), ApoE−/− mice under HFHC treated with NaCl (**B**) or EP80317 (**C**). HFHC diet increased BM thickness in ApoE−/− mice; this effect was prevented by CD36 stimulation using EP80317 (EP: 300 μg/kg, sc) (C,D). Scale bars: 1μm. RPE refers to retinal pigment epithelium, and Ch to choroid. Values are mean ± SEM; n=5/group. **p<0.05 compared to values without asterisks.

### CD36 stimulation prevents thickening of BM and preserves (in part) photoreceptor function

Additional experiments were conducted to determine if CD36 stimulation could prevent thickening of BM in HFHC-fed ApoE−/− mice. For this purpose animals were treated daily either with NaCl or the CD36 agonist EP80317 [36] from 8 to 18 weeks of age. HFHC diet significantly increased the thickness of BM in saline-treated ApoE−/− mice (Figure 4B,D), as previously reported [24]. Whereas, treatment of ApoE−/− mice with EP80317, which reduces plasma cholesterol levels [36], prevented the accumulation of sub-RPE deposits and thickening of BM (Figure 4C,D).

**Figure 5.**
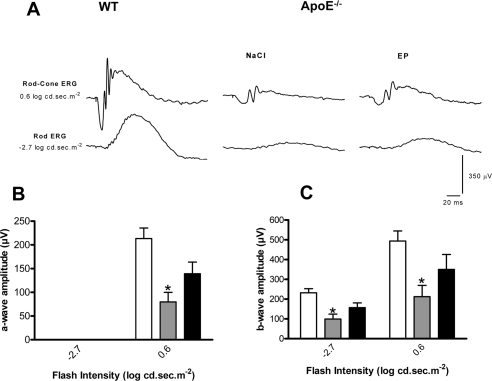
CD36 stimulation improves visual function in ApoE−/− Representative rod-cone (0.6 log.cd.sec.m^−2^) and rod (−2.7 log.cd.sec.m^−2^) electroretinographic tracings from WT and ApoE−/− under HFHC treated with NaCl or EP80317 (A). Amplitudes of the a-waves (rod-cone ERG) (B) and b-waves (rod and rod-cone ERGs) (C) were significantly reduced in ApoE−/− mice injected with NaCl (grey bars) compared to WT mice (white bars); EP80317 treatment significantly attenuated this decrease (black bars). Values are mean ± SEM; n=6/group. *p<0.05 compared to values without asterisks.

Along with BM thickening, ApoE−/− mice also present photoreceptor dysfunction [24,25], as evidenced with the significantly attenuated rod-cone a-wave amplitude of saline-treated ApoE−/− compared to WT mice (Figure 5A, B). Similarly, the significantly attenuated rod and rod-cone b-waves (−2.7 log cd.sec.m-2 and 0.6 log cd.sec.m-2, respectively) are also suggestive of inner retinal dysfunction in saline-treated ApoE−/− compared to WT mice (Figure 5A, C); treatment of ApoE−/− mice with EP80317 significantly attenuated the loss of the scotopic rod and rod-cone responses (Figure 5A-C).

## DISCUSSION

The molecular mechanisms that lead to lipid accumulation in BM are not well understood. Since lipids including oxidized sterols are believed to be an important component of sub-retinal debris [42,43], and CD36 is a major scavenger receptor of oxidized lipids [29,30] including in RPE [15], we surmised that CD36 modulates sub-retinal deposit formation. We found that oxLDL uptake in RPE is CD36-dependent in vitro and in vivo. We also showed that in animals deficient in CD36 on regular diet, oxLDL accumulated sub-retinally and was associated with BM laminar deposits analogous to those described in ApoE- and LDL receptor-null mice [19,23-24] fed on high fat high cholesterol diet. Moreover, stimulation of CD36 prevented accumulation of sub-retinal deposits and attenuated photoreceptor malfunction. Findings suggest that insufficiently prompt clearance of dietary lipids is associated with increased circulating levels of oxLDL and sub-retinal build-up of these lipids with other debris; the scavenger receptor CD36 contributes in maintaining integrity of BM.

Bruch's membrane (BM) is a thin layer of connective tissue located between RPE and choroid through which essential molecules for chorio-retinal homeostasis must be transported [44]. With age BM accumulate neutral lipids (triglyceride, esterified cholesterol, fatty acid) [7] and lipoprotein-like particles [6] comprised in laminar deposits, and lead to a decrease in permeability [45,46].

With time these BM deposits and drusen formations in the RPE/BM complex ultimately impact RPE and photoreceptor integrity [2,3]; these drusen are also composed of cellular debris, proteins such as complement [43] and β-amyloid [47-48], and lipids including esterified and unesterified cholesterol [8,49,50] and apolipoproteins (ApoE, B, A-I, C-I and C-III) [50-53]. Sub-retinal lipids originate in part from RPE [43,51] but also likely from plasma [8], consistent with findings in the present study. Accordingly, we found that CD36 expressing animals revealed RPE uptake of intravenously injected DiI-tagged-oxLDL, while CD36-null mice exhibited no such uptake from circulation (Figure 1).

Several receptors are potentially implicated in lipid transport in retina [27,28]. Mice deficient in LDL receptor or its ApoE ligand, display an atherosclerosis phenotype including high circulating levels of oxLDL [21], along with BM thickening, BM lipid deposits and RPE basal deposits [19,23-25]. Although debatable, an association between atherosclerosis and human AMD has also been described [54-56]. Our observations complement these findings, by showing that CD36 influences the clearance of oxLDL by RPE (Figures 1 and 3A), affecting (at least partly) sub-retinal structure; along these lines, CD36-null mice accumulated over time sub-retinal oxLDL with deposits (Figures 2 and 3D), while CD36 stimulation prevented the latter (Figure 4).

In summary, deficiency in uptake of oxidized LDL by CD36 expressed on the basolateral side of RPE [32] seems to contribute to age-related BM thickening; conversely, CD36 activation attenuates BM thickening and in parallel preserves visual function. In view of the marked paucity in effective therapeutic modalities for non-proliferative AMD, pharmacological modulation of CD36 activity may be a potential approach for this form of maculopathy.

## METHODS

### Animals

The ApoE−/− and ApoE/CD36 double deficient (ApoE−/−CD36−/−) mice, obtained as described previously [35], were housed at local animal facilities under 12 hours light-12 hours dark cycles and fed ad libitum with a normal (ND) or a high fat-high cholesterol diet (HFHC) (D12108, cholate, AIN-76A semipurified diet, Research Diets Inc., NewBrunswick, NJ). CD36−/− mice and their controls wild-type littermates (CD36+/+) were reproduced separately under ND unless otherwise indicated. Eight-week-old ApoE−/− mice were treated daily with EP803017 (300μg/Kg) [36] or vehicule (0.9% NaCl) by subcutaneous injections for a period of 10 weeks prior to sacrifice. All mice were sacrificed by carbon dioxide inhalation or by intraperitoneal injection of pentobarbital sodium overdose, prior to ocular enucleation. All experimental procedures were done in accordance with the Institional Animal Ethics Committee and the Canadian Council on Animal Care guidelines for use of experimental animals.

### Electron microscopy

Eyes were fixed for 1 h in 2.5% glutaraldehyde in cacodylate buffer (0.1 M, pH 7.4). After 1 h, the eyes were dissected, fixed for another 3 h, postfixed in 1% osmium tetroxide in cacodylate buffer, and dehydrated in graduated ethanol solutions. The samples were included in epoxy resin and oriented. Ultra-thin sections (80 nm) were contrasted by uranyl acetate and lead citrate and were observed with an electron microscope JEOL 100 CX II (JEOL) with 80 kV, and measurements of Bruch's membrane thickness were made on three representative animals.

### RPE primary culture

Eight at 12-day-old CD36+/+ and CD36−/− mice were sacrificed, enucleated and eyes were maintained at room temperature overnight in Dulbecco's Modified Eagle's Medium (DMEM, Invitrogen) and then incubated 30 min with 2 mg/ml trypsin/collagenase I at 37 °C. After trypsin inhibition with DMEM containing 10% fetal calf serum, the RPE layer was harvested. The RPE was plated in 8-wells labtek (Nunc) at a rate of RPE from one eye per well in DMEM containing 10% FCS, 1% penicillin/streptomy- cin. Cells were maintained for 7 days before the phagocytosis assay.

### Phagocytosis assay

Confluent RPE monolayers were challenged with DiI-oxLDL (Biomedical Technology Inc, MA, USA). When RPE reached 80% of confluence, cells were incubated with 30μg/ml DiI-oxLDL for 5 hours in DMEM containing 5% lipoprotein-deficient serum (Biomedical Technology Inc) at 37°C. After incubation, no-ingested DiI-oxLDL were removed by washing three times with PBS and cells were fixed for 10 min in paraformaldehyde 4%. The cells were then permeabilized for 30 min in 1% triton X-100, blocked with 1% bovine serum albumin in PBS for 30 min and incubated with 4', 6-Diamidino-2-phenyl-indole (DAPI) (1:4000, Sigma-Aldrich) for 5 min. The slides were mounted and observed with a Nikon Eclipse E800.

### Intravenous injection of DiI-oxLDL

CD36+/+ and CD36−/− mice (2-month-old) were anesthetized and intravenous injection of 100 μl of tag DiI coupled or not to oxLDL (Biomedical Technology Inc) were given in the tail vein. The mice were euthanatized 24 hours after injection and eyes were collected and fixed in 4% paraformaldehyde in PBS for 2 hours. The tissue was then mounted in OCT (Tissue Tek), cut in thin sections (10μm). Nuclei were labeled with DAPI (1:4000) and sections were mounted with Gelmount (Biomeda). Fluorescence was observed with a Nikon Eclipse E800.

### Immunofluorescence

Twelve-month-old CD36+/+ and CD36−/− mice eyes were fixed in paraformaldehyde 4% in PBS for 1 hour at room temperature and rinsed in PBS before embedded in OCT. Frozen transverse sections 10 μm thick were cut and permeabilized for 10 min in 1% Triton X-100. Postfixation was performed with methanol or ethanol, depending on the antibody used. Immunolabeling with primary antibodies (1:100) rabbit polyclonal oxLDL (RayBiotech Inc.) was performed overnight at room temperature. After washing in PBS, secondary antibodies coupled with Alexa Fluor 488 (1:100, Molecular Probes) were applied for 1 hour at room temperature. Nuclei were labeled with DAPI (1:4000) and sections were mounted with Gelmount. Fluorescence was observed with an Olympus BX51 microscope. All immunostaining were repeated at least three times, and staining without primary antibody served as negative controls.

### OxLDL ELISA

Ten- and 24-month-old C57Bl6/J mice (Jackson Laboratory) under ND and 12-week-old ApoE−/− mice under ND or HFHC diet and 12-week-old ApoE−/−CD36−/− under HFHC diet were anesthetized. Blood was drawn from the inferior vena cava mice using EDTA as an anticoagulant and butylated hydroxytoluene (20 μM final concentration) as antioxidant. Plasma was collected by centrifugation for 15 min at 1,000 g. Mouse plasma oxLDL level was determined by use of an ELISA kit (Accurate Chemical and Scientific Corp, USA).

### Electroretinography

Dark adapted scotopic full-field electroretinograms (ERG) (intensities:-6.3 log cd.sec.m^−2^through 0.6 log cd.sec.m^−2^) were obtained from 18-week-old ApoE−/− mice on HFHC diet treated or not with EP803017 and WT control-age matched mice as we described in detail [37-39]. Amplitudes of ERG a-wave and b-wave components were measured according to a method previously described [37,38]. Briefly, the amplitude of the a-wave was measured from baseline to trough, and the b-wave amplitude was measured from the trough of the a-wave to the peak of the b-wave.

### Statistical analysis

Data between groups (other than for ERG) were compared using non-parametric Mann Whitney U-test. For ERG data, 2-way repeated measures ANOVA (P < 0.05) with Bonferroni post-tests were used to compare WT mice to ApoE−/− mice, and to determine the effect of EP80317 treatment on the different parameters of the ERG as the repeated factor and treatment group as the independent factor. All analysis and graphic representations were performed with Prism software (version 4.0c; GraphPad Software); values are represented as mean ± standard error of the mean (SEM). P values were calculated for a confidence interval of 95%; hence P values of less than 0.05 were considered significant.
